# In vitro immunotherapy potency assays using real-time cell analysis

**DOI:** 10.1371/journal.pone.0193498

**Published:** 2018-03-02

**Authors:** Fabio Cerignoli, Yama A. Abassi, Brandon J. Lamarche, Garret Guenther, David Santa Ana, Diana Guimet, Wen Zhang, Jing Zhang, Biao Xi

**Affiliations:** ACEA Biosciences, San Diego, California, United States of America; Universite Paris-Sud, FRANCE

## Abstract

A growing understanding of the molecular interactions between immune effector cells and target tumor cells, coupled with refined gene therapy approaches, are giving rise to novel cancer immunotherapeutics with remarkable efficacy in the clinic against both solid and liquid tumors. While immunotherapy holds tremendous promise for treatment of certain cancers, significant challenges remain in the clinical translation to many other types of cancers and also in minimizing adverse effects. Therefore, there is an urgent need for functional potency assays, in vitro and in vivo, that could model the complex interaction of immune cells with tumor cells and can be used to rapidly test the efficacy of different immunotherapy approaches, whether it is small molecule, biologics, cell therapies or combinations thereof. Herein we report the development of an xCELLigence real-time cytolytic in vitro potency assay that uses cellular impedance to continuously monitor the viability of target tumor cells while they are being subjected to different types of treatments. Specialized microtiter plates containing integrated gold microelectrodes enable the number, size, and surface attachment strength of adherent target tumor cells to be selectively monitored within a heterogeneous mixture that includes effector cells, antibodies, small molecules, etc. Through surface-tethering approach, the killing of liquid cancers can also be monitored. Using NK92 effector cells as example, results from RTCA potency assay are very well correlated with end point data from image-based assays as well as flow cytometry. Several effector cells, i.e., PBMC, NK, CAR-T were tested and validated as well as biological molecules such as Bi-specific T cell Engagers (BiTEs) targeting the EpCAM protein expressed on tumor cells and blocking antibodies against the immune checkpoint inhibitor PD-1. Using the specifically designed xCELLigence immunotherapy software, quantitative parameters such as KT_50_ (the amount of time it takes to kill 50% of the target tumor cells) and % cytolysis are calculated and used for comparing the relative efficacy of different reagents. In summary, our results demonstrate the xCELLigence platform to be well suited for potency assays, providing quantitative assessment with high reproducibility and a greatly simplified work flow.

## Introduction

Immunotherapy is one of the most important paradigm shifts in the history of cancer treatment, where the exquisite specificity and potency of the immune system is unleashed to seek out and destroy different types of malignancies [1]. Immunotherapeutic approaches, including adaptive cell therapies, checkpoint inhibitors, oncolytic viruses, and Bispecific T cell Engagers (BiTEs) are displaying high efficacy in a growing number of contexts. However, the field continues to be plagued by wide variation in the degree and durability of patient responses and side effects, and numerous cancers remain totally refractory to immunotherapy intervention [2]. To accelerate the pace at which immunotherapeutics are designed, optimized, and translated into clinical applications, new tools are needed which can provide during the early stages of development and manufacturing, both mechanistic insights and accurate prediction of efficacy once introduced to the patient.

When developing and manufacturing biomolecule and cell-based products for immunotherapy, potency assays are employed to evaluate critical quality attributes (CQA) of the product. Any assay used for assessing CQAs must have the following characteristics: (1) high sensitivity and specificity, (2) quick turnaround, (3) accuracy, (4) representativeness of the mechanism of action, (4) coverage of all product constituents, (5) reproducibility, and (6) predictivity of clinical efficacy [3–6]. While a single potency assay may not necessarily cover all these important attributes, ultimately a multitude of different assays may need to be implemented to cover the most important aspects of the immunotherapy during R&D and manufacturing processes. The potency assays currently employed in immunotherapy research and development include a vast array of techniques, ranging from *in vivo* animal models to biochemical and cell-based assays [3, 5, 7]. Using the appropriate potency assay in the proper context and understanding its advantages and limitations is critical for obtaining accurate results. Most cancer immunotherapy approaches, including cancer vaccines, BiTEs, immune checkpoint modulators, tumor infiltrating lymphocytes (TILs), and chimeric antigen receptor T cells (CAR-Ts), aim to unmask the cytotoxic effector function of the innate and adaptive immune systems to recognize and destroy malignant cells. Accordingly, it is important that any potency assay used for evaluating these immunotherapy products is able to recapitulate the dynamic and complex interactions between the target cell and the effector molecule or cell.

A number of potency assays which either directly measure the cytotoxic activity of the effector cells or through certain surrogates markers such as cytokine release, have been described. Among these, the chromium 51 (^51^Cr) release assay is considered the gold standard for monitoring immune cell-mediated killing and continues to be used extensively in the field [8]. While chromium 51 release is a fairly sensitive assay, there are certain drawbacks such as the use of radioactive material and the limited time to conduct the assay due to high spontaneous isotope leakage from the target cells [9]. In addition, due to the time constraint in the assay, typically very high effector to target (E:T) ratios must be used in order to observe target cell killing. The use of such high E:T ratios and short time assay windows may diminish the physiological relevance and specificity of the assay, precluding the fundamental serial killing activity of an effector cell encountering multiple tumor cells once infused into the patient [10]. In addition to the chromium 51 assay, other commonly used assays include flow-cytometry based detection of key biomarkers [11], luciferase reporter assays [12, 13], ELISpot assays [14, 15], and LDH release. The major drawbacks of these assays are that: (1) they typically involve labeling or engineering either the target or effector cells, which can limit their utility in terms of the types of effectors or target tumor cell models that can be used; (2) the end point nature of these assays which limits the derivation of kinetic information; (3) the incompatibility with other types of orthogonal assays which can provide additional mechanistic information; and (4) they are labor intensive and complex to set up, which effectively limits the throughput of the assay.

**Fig 1:**
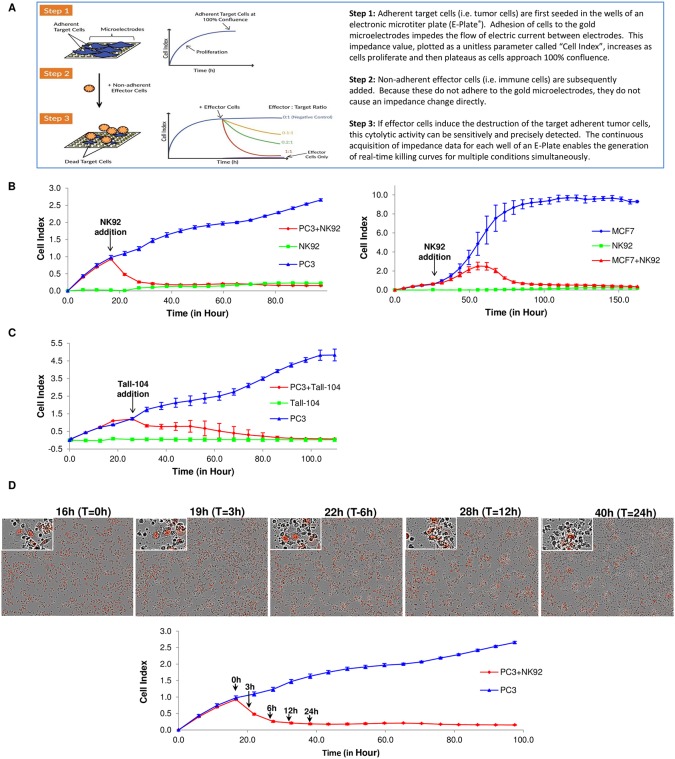
Working principle of the xCELLigence RTCA system and result from NK92 and TALL-104. (A) Working principle of xCELLigence impedance technology applied to immunotherapy monitoring. The xCELLigence RTCA label-free technology monitors cell number by changes in impedance measured through gold electrodes embedded in proprietary E-Plates. When seeded alone, target adherent cancer cells proliferation rate is registered as increase in the impedance-related Cell Index (CI) parameter over time. Effector non-adherent immune cells produce small baseline level signal due absence of tight surface adhesion over the gold electrodes. When immune cells are added to adherent target cells, their cytolytic activity causes the adherent cells to round up and detach, consequently reducing CI value. (B) Impedance monitoring is validated using NK92 as effector cells over nuclear red-labeled PC3 prostate cancer cells (left) or MCF7 breast cancer cells (right) as targets. When seeded alone, target cells adhere to the plate and proliferate, increasing the CI readout (blue lines). NK92 effector cells seeded alone caused only a small increase in the CI value over the initial background measurement (green lines). When added to target cells, NK92 cause cell cytolysis and subsequent progressive decrease in CI (red lines). Y-axis is the normalized cell index generated by the RTCA software and displayed in real time. X-axis is the time of cell culture and treatment time in hour. Mean values of the CI were plotted ± standard deviation. Time interval is 6 hours for all the figures unless indicated. (C) The same PC3 target cells are treated with a cytotoxic T cell line (Tall-104), showing the suitability of the technology to use different effector cell types. (D) Images taken at different time points after NK92 addition show correlation between CI drop (red line in the plot), reduction in target cells number (red nuclei PC3 in the images) and changes in cell morphology/adhesion in apoptotic cells (red nuclei in images enlargements).

To overcome the above limitations we have adapted a non-invasive, label-free, and real-time cellular impedance monitoring technology (xCELLigence) to measure the potency of biologics and immune cell mediated cytotoxicity. We have also developed a new software suite to automatically convert the impedance parameter to % cytolysis and to other kinetic relevant parameters like the killing time (KT). The xCELLigence platform utilizes gold microelectrodes embedded in the bottom of microtiter wells to monitor the status of adherent cells, or suspension cells which have been tethered to the plate bottom [16–20]. The basic assay principle is based on impedance measurements through the surface of gold electrodes where attached cells act as insulators, impeding the flow of an alternating microampere electric current between electrodes. This impedance signal is measured automatically, at a frequency defined by the user (every 10 seconds, once per hour, etc.), and provides an extremely sensitive readout of cell number, cell size, and cell-substrate attachment strength. In contrast to surface-attached cancer cell targets, immune effector cells are non-adherent and therefore do not directly affect the impedance signal; however their cytotoxic activity can be detected through the reduction of the target cancer cells number. Because of this property, the cytolytic activity of NK cells, T cells, CARTs, oncolytic virus, checkpoint inhibitors, bispecific antibodies, BiTEs, etc. can be selectively monitored in real-time (Fig 1A).

In order to thoroughly evaluate and validate the utility of this real-time potency assay, herein the xCELLigence instrument is used for conducting diverse killing assays involving a variety of cancer target cells and immune effector cells. The resulting real-time impedance traces and their associated killing kinetics, as well as quantitative parameters such as EC_50_ and KT_50_ are used to derive mechanistic conclusions and potency ranks. Collectively, these results indicate that real-time potency assays using the xCELLigence system is an effective and efficient tool for both basic and applied immunotherapy research as well as monitoring CQA of various immunotherapies during manufacturing.

## Materials and methods

### Cell lines and reagents

All cell lines were obtained from ATCC. PC3 and MCF7 were cultured in RPMI 1640 (ATCC) with 10% FBS (HyClone) and Pen/Strep (Gibco). PC3 is a human prostate cancer cell line while MCF7 is human breast cancer cells derived from metastatic site. NK92, a natural killer cell line derived from a patient with malignant non-hodgkind’s lymphoma, is cytotoxic to a wide range of other malignant cells and was cultured in Alpha Minimum Essential medium without ribonucleosides and deoxyribonucleosides but with 2 mM L-glutamine and 1.5 g/L sodium bicarbonate (Sigma). To make the complete growth medium, the following components were added: 0.2 mM inositol, 0.1 mM 2-mercaptoethanol, 0.02 mM folic acid, 12.5% horse serum, and 12.5% fetal bovine serum. IL-2 (Peprotech) was used at a concentration of 200 U/mL during cell culture but not in the experiments. TALL-104 (ATCC) is a cytotoxic T lymphocyte cell line that kills tumor cells in a MHC independent manner, established from the peripheral blood of a child in relapse with T-ALL. It is cultured in conditions recommended by ATCC. To make the complete basal growth medium, the following components were added to Iscove’s Modified Dulbecco’s Medium: 100 units/mL recombinant human IL-2, 2.5 microgram/mL human albumin, 0.5 microgram/mL D-mannitol, and fetal bovine serum to a final concentration of 20%. Raji lymphoma cells were cultured in RPMI1640 with 10% fetal bovine serum. PC3 nuclear red cells were generated by transducing PC3 cells with the NucLight Red Lentivirus (Essen Biosciences) and selected with puromycin. CD19 targeting CAR-T and mock cells were provided by ProMab Biotechnologies. Staphylococcal enterotoxin B (SEB) was from Toxin Technology, Inc. FL. Anti-EPCAM & Anti-CD3e Bispecific Antibody (scFv), BiTE, Recombinant and Anti-human PD-1 antibody (A2002, Nivolumab) were obtained from G&P Biosciences and Selleck Chemicals.

### Immune cell isolation

Peripheral Blood Mononuclear Cells (PBMCs) were obtained from commercial provider, iXcells Biotechnologies, based on the protocol approved by their Institutional Review Board (IRB). The protocol to freshly isolate PBMCs from healthy donors is using Ficoll-Paque Plus (GE Healtcare) according to manufacturer’s protocol. Briefly, freshly collected blood was diluted with two-fold PBS (Ca/Mg, -/-). The diluted blood was then gently loaded on top of 15 mL Ficoll-Pague Plus in a 50 mL tube. According to the manufacturer’s protocol, the tube was spun at 350xg for 35 minutes at room temperature. The supernatant was removed and the cells collected with another spin at low speed, 400xg for 5 minutes. Once collected, the cell pellet was subjected to red blood cells lysis by applying Red Blood Cell Lysis Buffer (Biolegend) for 5 minutes. The remaining cells were then washed 2–3 times with PBS (Ca/Mg, -/-). Finally, the PBMCs were collected and resuspended in RPMI 1640 with 10% FBS and Pen/Strep. Once PBMCs were isolated, they were either cultured briefly in the above medium or were added to target cells immediately.

### Real-time potency assessment using the xCELLigence RTCA

The xCELLigence RTCA MP instrument (ACEA Biosciences) was utilized for all impedance experiments. First, 50 uL of target cancer cell culturing media was added to each well of 96 well E-Plates (ACEA Biosciences) and the background impedance was measured and displayed as Cell Index[16, 19, 21]. Dissociated adherent target cells were seeded at a density of 10,000 (PC3) or 40,000 (MCF7) cells/well of the E-Plate in a volume of 100 uL and allowed to passively adhere on the electrode surface. Post seeding, the E-Plate was kept at ambient temperature inside a laminar flow hood for 30 minutes and then transferred to the RTCA MP instrument inside a cell culture incubator. Data recording was initiated immediately at 15 minute intervals for the entire duration of the experiment. At the time treatment was applied, data acquisition was paused, 100 uL of media was removed from each well and effector cells were added at different effector to target (E:T) ratios in a volume of 100 uL. In the experiments where BiTEs were used, 20 uL of antibody solution was added immediately prior to addition of effector cells. Effector cell only controls, target plus Mock effector controls, and full lysis controls, where a final concentration of 0.25% Triton X-100 (Sigma) was added to target cells, were also set up. The E-Plate was kept for 30 minutes in the laminar flow hood before resuming the experiment. Changes in impedance were reported as Cell Index (CI) and Normalized Cell Index (NCI), which have been described previously [16, 20, 21]. After normalizing the data to account for “target cell alone” and “effector cell alone” controls, parameters such as % Cytolysis and KT_50_ were determined using the xIMT software (ACEA Biosciences). The % Cytolysis plot utilizes a Target Alone Control and a Normalization Time to calculate the % of Cytolysis at every time point. For each well, the % Cytolysis utilizes the Normalized Sample Cell Index and the Normalized Average Target Alone Control according to the following equation:

$$\%\cdot Cytolysis=[1-\frac{CI_{ti}/CI_{nml\_time}}{\left(\bar{CI_{T\text{arg}etAlone\cdot ti}/CI_{T\text{arg}etAlone\cdot nml\_time}}\right)}]\times 100$$

Where:

*CI*_*ti*_ is the average Cell Index between replicate wells at the time *ti*

*CI*_*nml_time*_ is the average Cell Index between replicate wells at normalization time

*CI*_*TargerAlone ti*_ is the average Cell Index between replicate target alone control wells at the time *ti*

CI_Targetalone nml_time_ is the average Cell Index between replicate target alone control wells at normalization time

### Suspension target cells killed by NK92 and CAR-T effector cells

To adapt the xCELLigence E-Plate for analyzing killing of suspension target cells, the E-Plate wells were incubated with the appropriate tethering reagent (B cell killing kit, ACEA Biosciences), at a concentration of 4 μg/mL, for 3 hours at 37°C. After washing the wells with tethering buffer, Raji lymphoma B cells were seeded at a density of 40,000–60,000 cells per well. The E-Plate was then kept at ambient temperature inside a laminar flow hood for 30 minutes and then transferred to the RTCA MP instrument inside a cell culture incubator. Impedance measurements were started immediately to monitor cell attachment and growth. The following day, NK92 effector cells were added on top of the Raji cells at different E:T ratios. Each condition was tested in triplicate. An “effector cell only” control and a “full lysis” control were included. After placing the E-Plate back inside the xCELLigence instrument, data acquisition was resumed to monitor NK92 cytotoxic activity based on the viability of the surface attached Raji cells, as reflected by Cell Index values. The xIMT software was used to plot the percentage of cytolysis and calculate parameters such as KT_50_. A parallel experiment was set up in which the Raji cells were incubated with NK92 cells in suspension only. Then cells were collected and subjected to FLOW analysis (NovoCyte, ACEA Biosciences). Using CD56 (Clone5.1H11, Biolegend) as marker for NK92 cells, CD19 (Clone HIB19, Biolegend) as a marker for Raji cells, and 7-aminoactinomycin D (7-AAD) as a marker of membrane integrity/cell death were employed to measure killing efficacy. Experiment with CAR-T were conducted in RPMI1640 medium with 10% FBS without IL-2.

### Enhanced PC3 cell killing by PBMCs using anti-PD-1 antibody

PC3 cells were seeded in an E-Plate at a density of 5,000 cells per well. Fresh PBMCs from healthy donors were counted and added on top of the attached PC3 cells at different E:T ratios. Along with the PBMCs, Staphylococcal enterotoxin B (SEB) and anti-PD-1 antibody were also added. Cell Index and % Cytolysis were determined using the xIMT software.

### Flow cytometry analysis

The percentage of cytolysis determined by xCELLigence was confirmed using flow cytometry, by tracking apoptosis marker expression. Briefly, duplicate E-Plates were set up simultaneously with one plate placed on the RTCA instrument and the other plate treated exactly the same way but used for flow cytometry analysis. For flow cytometry analysis, cells were trypsinized from quadruplicate wells and combined together. Dead floating cells were also collected and added to the trypsinized samples. After washing the collected cells with PBS (Gibco) cells were stained with annexin V FITC (Biolegend) and 1 ug/mL DAPI for 20 minutes according to the manufacturer’s protocol. Stained cells were analyzed using the NovoCyte flow cytometer (ACEA Biosciences) and data analysis was performed using the NovoExpress software (ACEA Biosciences). Nuclear red positive PC3 cells and annexin V/DAPI positive apoptotic cell populations were gated based on single cell type staining controls.

### Image acquisition

Impedance data were compared to cell number and morphological changes using an IncuCyte Zoom automatic microscope (EssenBio) and standard 96 well cell culture plates. Images were acquired using bright field and red fluorescent channels.

### Statistical analysis

Data generated from the xCELLigence instrument are reported as the average and standard deviation of well replicates. Statistical significance was determined with one way ANOVA analysis using GraphPad Prism 6. Differences were considered Not Significant (NS) when P>0.05, or significant when P<0.05 (*); P<0.01 (**); P<0.001 (***) and P<0.0001 (****). The correlation coefficient was calculated by CORREL function in Microsoft Excel 2010.

## Results

### Monitoring effector-mediated cytolysis of target cancer cell lines using xCELLigence real-time cell analysis

As described in Fig 1A, the RTCA system utilizes cellular impedance readout to monitor real-time changes in cell number, cell size, and cell-substrate attachment strength as one overall parameter named Cell Index (CI) to reflect the viability of target cancer cells. In order to validate the utility of this approach as a potency assay for immune cell-mediated cytolysis of target cells, multiple cancer cell lines were treated with different types of effector cells. First, PC3 prostate cancer cells which had been engineered to express a nuclear far-red fluorescent protein (mKate2) were cultured in E-Plates and their proliferation monitored using xCELLigence (Fig 1B, left panel). The day after seeding the PC3 target cells, the human-derived natural killer (NK) cell line NK-92 was added to the wells at an E:T ratio of 2.5:1 and the viability of the target PC3 cells was monitored. PC3 cells treated with effector cells growth media alone was set up as a negative control. To determine whether the NK-92 cells were able to generate an impedance signal, a separate control involved the addition of NK-92 cells to wells that did not contain any PC3 target cells. As expected, since NK-92 cells are naturally not adherent by themselves, these cells displayed a negligible impedance signal. The addition of NK-92 cells to PC3 cells caused an immediate and time-dependent decrease in CI. Roughly 20 hours post addition of effector cells the PC3 signal dropped to the same level of the NK-92 alone background control, which is consistent with complete cytolysis of the target cells (green line in Fig 1B, left panel).

Similar results were obtained when MCF7 target cells (Fig 1B, right panel) were treated with NK-92 cells at an E:T ratio of 1:1. Note, however, that the attachment, growth, and kinetics of cytolysis are different for MCF7 cells compared to PC3 cells (Fig 1B, left). The different kinetics of CI reduction shortly after NK92 addition may also indicate different sensitivity between the two cell lines in response to the NK-92 lytic activity. Consistent with our findings, a previous study showed PC3 cells to be more sensitive than MCF7 cells to cytolysis by NK-92 cells [22]. In addition to NK-92 cells, TALL-104, a human leukemic T cell line, was also used against PC3 target cells. As shown in Fig 1C, TALL-104 addition to PC3 resulted in a time-dependent decrease in CI, reaching the value of the effector alone background control ~60 hours post treatment. Similar to NK-92, addition of TALL-104 to empty wells resulted in a very small increase in CI. For both type of effector cells, the negligible background signal indicates that within the context of the assay the CI reflects the status of the target cells.

The reduction in CI value after addition of effector cells most likely reflects the loss of viability of target cells [13, 23, 24]. In order to determine if the drop in CI reflects loss of viability and morphological changes related to target cells, a parallel experiment with PC3 target cells and NK-92 effector cells was set up in standard 96 well microtiter plates and images were acquired at different time points utilizing an automatic microscope imaging platform. PC3 cell number and morphology were monitored using bright field and the red nuclear fluorescence (Fig 1D, top). Images acquired at different time points show the progressive reduction in PC3 cell number and profound changes in cell morphology that are indicative of apoptosis, including loss of cell membrane integrity, cell shrinkage, and detachment (enlarged inserts). Assessment of cell images by microscopy demonstrate that the kinetics of cell death correlates very well with the CI drop rate observed using xCELLigence (Fig 1D, bottom). Collectively, the above data confirm that the drop in CI is indicative of *bona fide* cell death, and demonstrate the efficacy of using xCELLigence for dynamically monitoring immune cell mediated killing of target cancer cells.

### Real-time cytolysis potency assays and derivation of KT_50_ values

**Fig 2:**
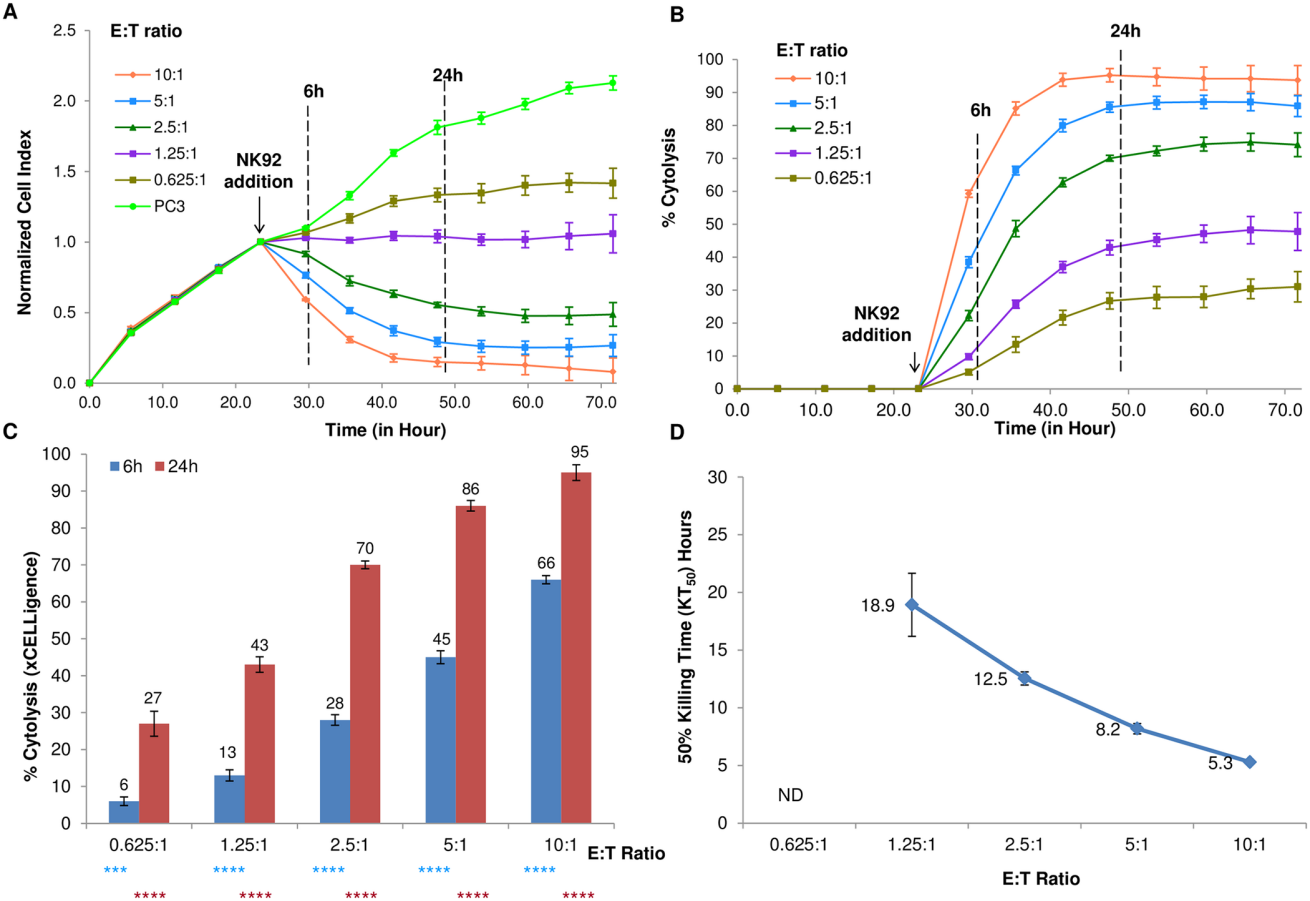
Parameters of % cytolysis and KT_50_ determined through impedance measurement. (A) Cell Index plot for nuclear-red labeled PC3 target cells treated with different E:T ratios of NK92 cytolytic cells. Samples have been internally normalized for the Cell Index value measured before NK92 addition (Normalized Cell Index plot). (B) The Cell Index plot is converted to a % Cytolysis plot by the xCELLigence Immunotherapy Software (xIMT). (C) % Cytolysis measured at 6 and 24 hours after NK92 addition for the different E:T ratios. One way ANOVA result indicates significant difference between individual treatment and control at 6 hours (light blue) and at 24 hours (red); (*** p< 0.001) and (**** p<0.0001). (D) 50% Killing Time (KT_50_) for the same E:T ratios in (C). ND: Not Detected.

In order to assess cytolysis of target cells as a function of time and effector cell number using the RTCA potency assay, nuclear red PC3 cells were treated with NK-92 at E:T ratios ranging from 10:1 to 0.625:1 using quadruplicate wells. As shown in Fig 2A, the addition of NK-92 to PC3 cells was followed by a time-dependent reduction in the CI values. As expected, the extent of CI drop is proportional to the number of NK-92 cells added. While CI drop after effector addition directly correlates with cell viability, it can be readily converted to percent cytolysis through mathematical calculations that take into account the signal from the target cells alone control, as described in the materials and methods section. As shown in Fig 2B, percent cytolysis increases in a time and E:T ratio-dependent manner. For several of the E:T ratios tested, the % cytolysis reach a plateau after 50 hours that is lower than 100% indicating incomplete lysis by NK92. We speculate that in conditions of low E:T ratios the effectors cells are a limiting factor and cannot kill all the target cells which continue to proliferate. It is possible that after a certain time the two cell populations reach an equilibrium in cell number that is reflected in the constant level of the signal. Fig 2C shows the extent of NK-92-mediated cytolysis of PC3 cells at different E:T ratios either 6 or 24 hours after NK-92 addition.

An advantage of continuous impedance-based monitoring is that the time dependency of cytolysis is captured at high frequency of measurements (e.g. 15 min intervals) which can be challenging with other approaches. As consequence, kinetic parameters that encompass the temporal information can be effectively derived. One example is the KT_50_ parameter, which represent the time required to reach 50% cytolysis at a given E:T ratio or other experimental conditions. As expected, there is an inverse correlation between the E:T ratio and the KT_50_ value observed, with smaller KT_50_ value indicating more efficient killing kinetic (Fig 2D). At E:T ratio of 0.625:1, 50% cytolysis was not achieved within the time frame of the assay and, accordingly, a KT_50_ for this condition could not be determined. The parameter of KT_50_ is complementary to the percent cytolysis parameter; while the latter shows the potency of a specific condition at given time point, the former allows analysis in the temporal dimension and effectively ranks the treatments according to the rate of cell killing.

**Fig 3:**
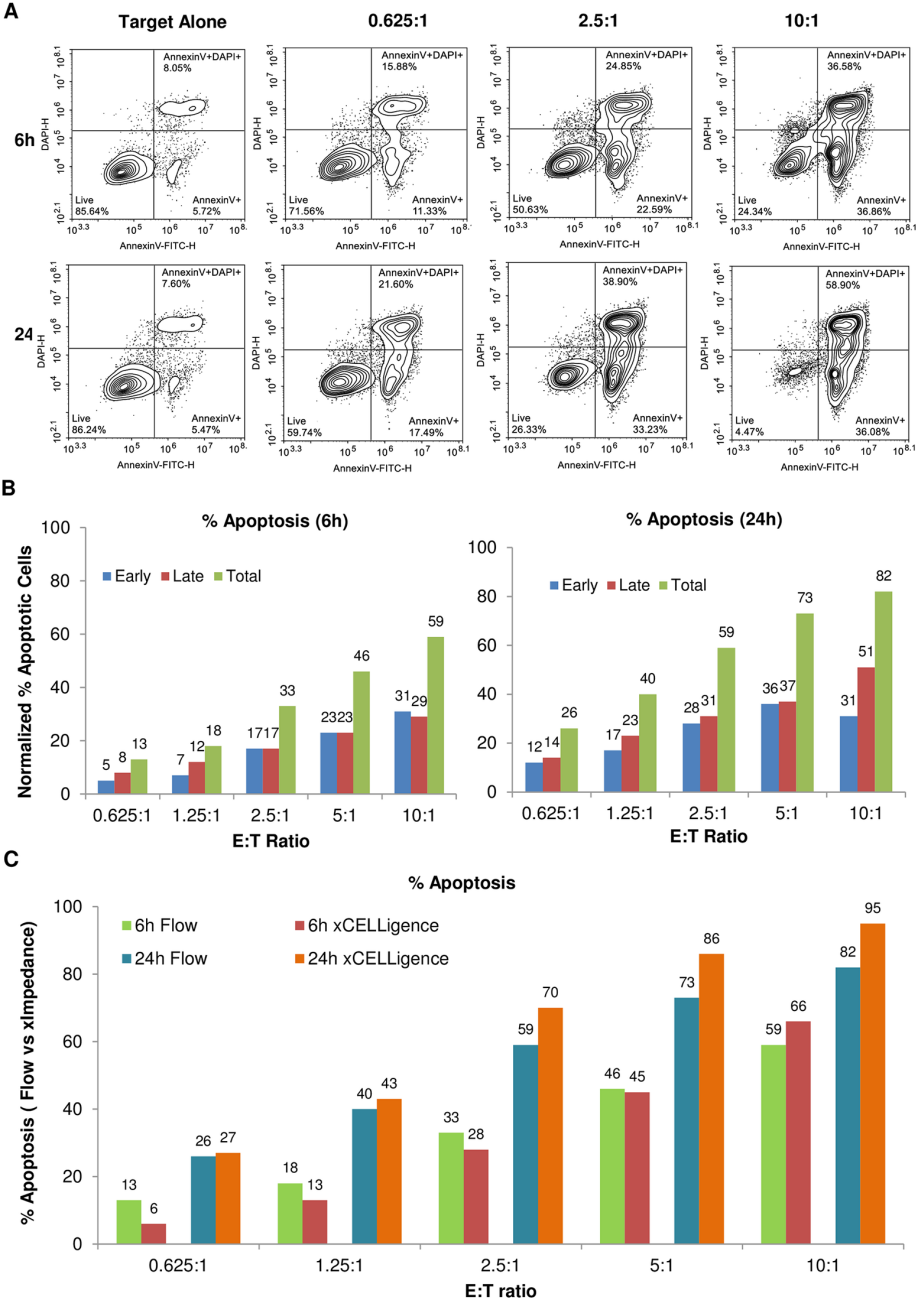
Correlation between % cytolysis determined through impedance measurement and flow cytometry. (A) Replica plates for the same experiment in Fig 2 has been collected and analyzed by Flow Cytometry. Nuclear red-gated PC3s show ratio and time dependent increase of early apoptotic (annexin V+, DAPI-; bottom right of each plot), and late apoptotic (annexin V+, DAPI+; upper right of each plot) cells. (B) Charts show the % apoptotic cells for the flow data. (C) Total apoptosis measured by flow cytometry is similar to the results of impedance analysis.

Despite the fact that the correlation between CI drop and cell killing is evident, a key question is how well the % cytolysis determined through impedance readout matches the results of other techniques that monitor marker expression and membrane integrity. To address this issue, the experiment described in Fig 2A was performed in parallel with a second plate where the extent of apoptosis of target PC3 cells was evaluated using flow cytometry at 6 and 24 hours post effector cells addition. Samples were stained for annexin V and DAPI to identify early and late apoptotic cells, respectively, within the nuclear red gated PC3 cell population (Fig 3A). Density plots at 6 hours post effector cells addition show a progressive increase in early (annexin V^+^, DAPI^-^) and late (annexin V^+^, DAPI^+^) apoptotic cells with increasing E:T ratio, reaching a maximum value of 31.5% late apoptotic cells and 46% early apoptotic cells at an E:T ratio of 10 (Fig 3B, left panel). The percentage of cells undergoing late and early apoptosis was further increased when the cells were analyzed 24 hours post effector cell addition, reaching 44.6% and 50.3% respectively at an E:T ratio of 10:1 (Fig 3B, right panel). The flow data for early, late, and total apoptotic cells are summarized in Fig 3B, highlighting a prevalence of early apoptotic cells over late apoptotic cells at both 6 and 24 hours. Importantly, the combined apoptotic cells from the flow data are in strong agreement with the % cytolysis measured by the xCELLigence RTCA potency assay (Fig 3C). The total apoptotic rate correlation coefficient between xCELLigence and flow at 6 and 24 hours, including all the different E:T ratios examined, are 0.9959 and 0.9987 respectively. Taken together, the flow data support the conclusion that the xCELLigence assay accurately reflects the number of dying target cells in the context of cell-mediated cytolysis. Furthermore, the close correlation of the impedance data and annexin V staining data suggests that once a target cells initiates the apoptotic process, rearrangements in cytoskeleton and membrane structure are immediately registered as changes in impedance [16, 17, 20, 21], thus identifying Cell Index decrease as another early marker of apoptosis in this potency assay.

### Monitoring the potency of Bi-specific T-cell Engagers (BiTEs) using xCELLigence

**Fig 4:**
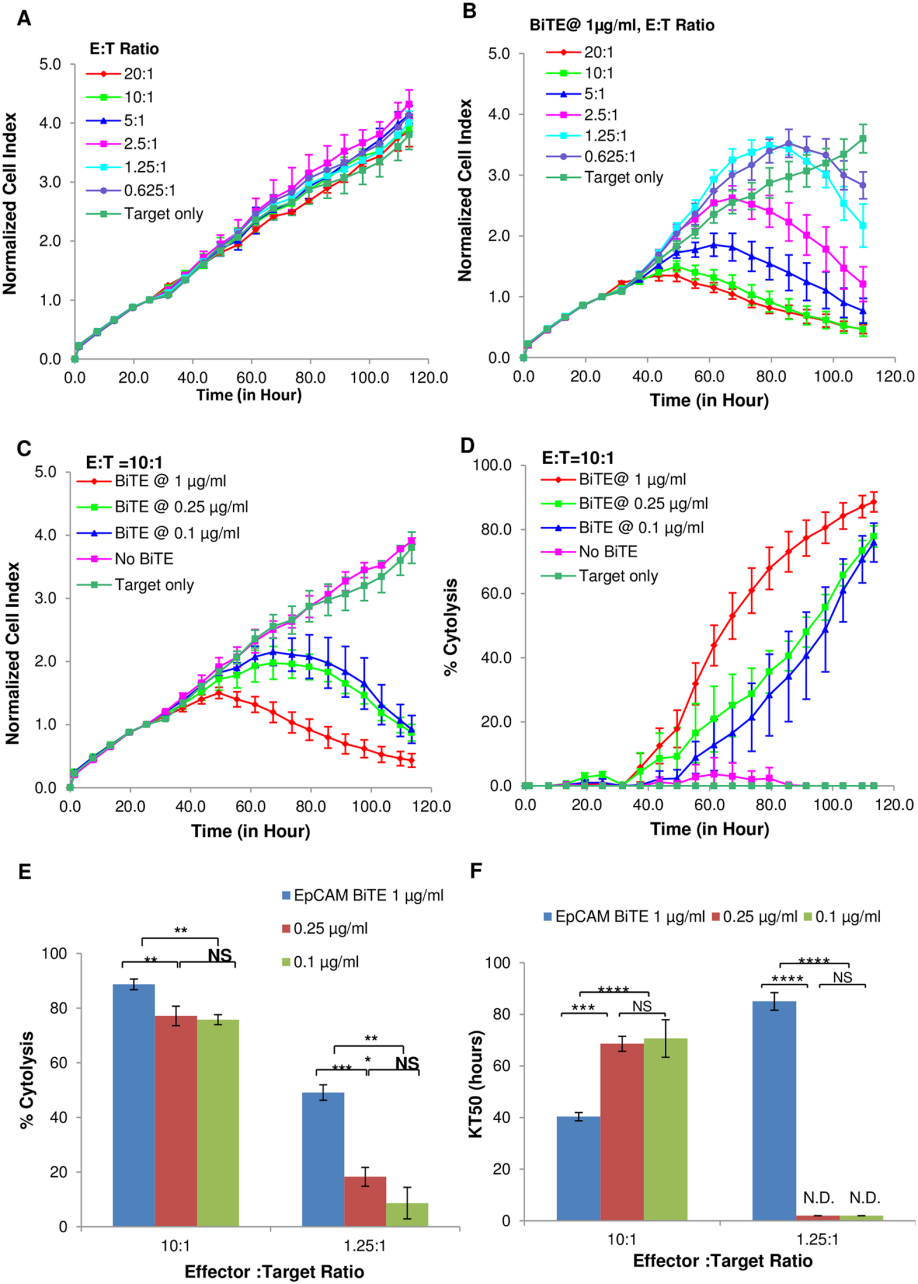
Impedance assessment of BiTE-mediated cytotoxicity. (A) Normalized Cell Index plot for PC3 target cells incubated with PBMCs at different E:T ratios without BiTE. (B) Same E:T ratios as (A) but with 1 μg/ml anti-EpCAM/CD3 BiTE. (C) At E:T ratio of 10:1, different BiTE concentrations resulted in varied dynamic cytolysis of the target cells. (D) Same result from (C) showed as % cytolysis plot. (E) Example of BiTE concentration depended % cytolysis from E:T ratio 10:1 and 1.25:1. (F) KT_50_ comparison for result from (E). Significant analysis performed by one way ANOVA. (*** p< 0.001),); (** p< 0.01);); (* p< 0.05);); (NS Not Significant); (ND Not Detected).

Having validated real-time potency assays in the context of constitutively active cytolytic effector cell lines, we next used this technology to study cell killing mediated by Bispecific T cell Engagers (BiTEs), which are a class of immunotherapy reagents that redirect the host’s immune system to recognize and destroy tumor cells and are currently being evaluated in clinical trials [25, 26]. These engineered artificial antibodies contain two high affinity recognition domains. While one domain binds a specific antigen on the surface of cancer cells, the other domain binds the T cell receptor, thereby directly linking effector cells to target cells. PC3 target cells were treated with freshly isolated Peripheral Blood Mononuclear Cells (PBMCs) in the presence or absence of a BiTE that targets EpCAM, a surface protein expressed in many carcinomas and currently being evaluated as a therapeutic target in several clinical trials [27, 28]. At the E:T ratios tested in this experiment, in the absence of the BiTE the PBMCs displayed no cytolytic activity (Fig 4A). However, the presence of 1 μg/mL EpCAM BiTE improved the cytolytic efficacy of the PBMCs, with CI decreasing in a manner proportional to the amount of PBMCs in the wells (Fig 4B). EpCAM BiTE by itself did not have an impact on PC3 cell viability (Data not shown), indicating that the target cell killing is occurring through BiTE-mediated tethering of effector and target cells. Next, the dose-dependency of the EpCAM BiTE was evaluated against PC3 cancer cells at an E:T ratio of 10:1 using the CI parameter (Fig 4C) and % cytolysis (Fig 4D). At the highest EpCAM BiTE concentration cytolysis occurs earlier, and to a much greater extent, than observed at lower concentrations. Interestingly, the data clearly show larger differences in cytolysis efficiency when less effector cells were used (Fig 4E). Furthermore, KT_50_ analysis shows that killing is still efficient at lower E:T ratios, as long as sufficient amount of EpCAM BiTE is present (Fig 4F).

Taken together, the above data demonstrate the xCELLigence assay to be effective for analyzing BiTE potency in a quantitative, dose and time dependent manner. More generally, the experiments with the EpCAM BiTE demonstrate the ability of xCELLigence to simultaneously analyze killing efficiency across multiple conditions and over broad assay windows, making the process of assay optimization both rigorous and efficient.

### Development of real-time potency assays for cancer cells originating from liquid tumors

**Fig 5:**
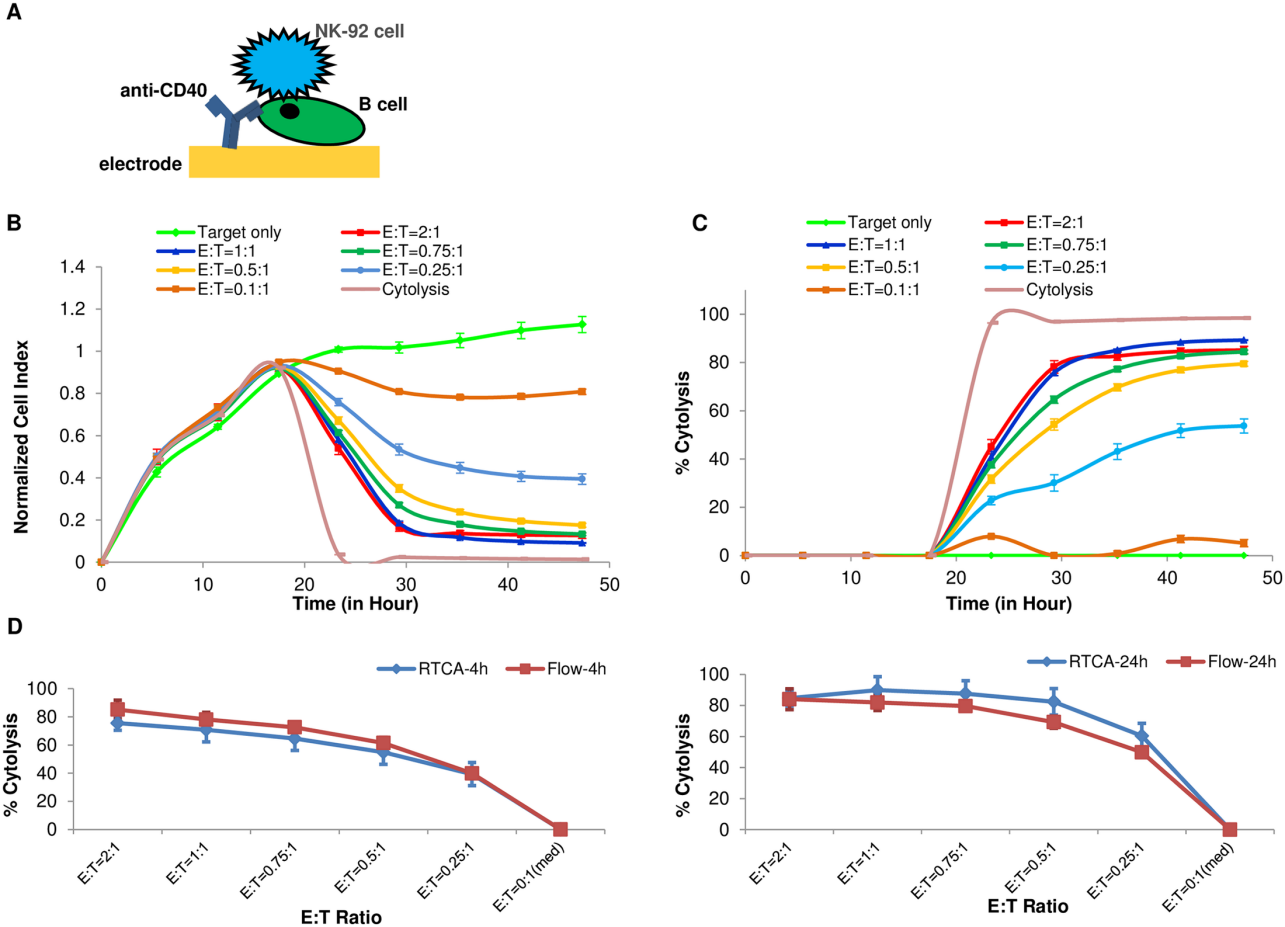
Adaptation of the xCELLigence killing assay to suspension B cells. (A). Illustration of B cell attachment through anti-CD40 antibody on the surface of the gold sensor embedded in the well. (B). Raji, 60,000 cells per well, were seeded on the E plate and NK92 cells were added to the well at different E:T ratios. Cytolysis and target cells only are used as positive and negative controls for cyutolysis. (C). Same data as (B) are plotted by xIMT software and displayed as % cytolysis. (D). Comparison of the % cytolysis of Raji cells at different E:T ratios measured by RTCA and flow cytometer at 4 hours (left, correlation coefficient R = 0.9967) and 24 hours (right, R = 0.9903).

To date, the most successful immunotherapies are those targeting blood-borne tumors such as acute lymphocytic leukemia. We therefore sought to adapt the xCELLigence potency assay for *in vitro* assessment of immunotherapies targeting tumor cell lines originated from liquid tumors. We speculated that if suspension tumor cells or cell lines could be immobilized on the electrode surface of E-Plates through antibody-mediated tethering, liquid tumor cytolysis assays could be carried out in a manner similar to adherent tumor cell targets. To achieve this, E-Plate wells were coated with an anti-CD40 antibody that selectively binds leukemic or lymphoma B cell lines (Fig 5A). Using this approach, one common lymphoma cell line, Raji can indeed be tethered to E-Plate electrodes, giving rise to a robust impedance signal (Fig 5B, target only). On the second day after Raji cells seeding, NK-92 cells were added at different E:T ratios, resulting in a dose-proportional decrease in Cell Index (Fig 5B) and % cytolysis(Fig 5C). For the assay to be accurate, it is important that the tethering antibody is selective for the target cells, thereby precluding any impedance signal derived from the effector cells. Indeed, addition of 60,000 NK-92 effector cells (which lack the CD40 receptor) to wells coated with the anti-CD40 antibody produced a CI signal of just 0.15, which is very close to background impedance signal(data not shown). In order to verify that surface-tethering Raji B cells does not perturb the extent of cytolysis by NK-92 cells, a parallel cytolysis assay was performed in solution (i.e. without tethering) and apoptosis was monitored at 4 or 24 hours using flow cytometry. As shown in Fig 5D, the extent of cytolysis observed in solution correlated very well with the extent of cytolysis determined using the xCELLigence assay; the correlation coefficients at 4 and 24 hours were 0.9967 and 0.9903, respectively. Flow cytometry comparison also showed no differences in apoptotic cells between tethered and non-tethered cells (Data not shown).

One of the main advantages of the xCELLigence system for conducting potency assays especially for screening purposes is the simplicity and rapidity of setting up and analyzing the data. To evaluate the suitability of real-time potency assays for screening purposes we tested the robustness of the Raji/NK-92 killing assay in three different experiments, using a larger number of replicates and comparing % Cytolysis and the Z-factor at 4 and 24 hours after effector addition. Z-factor is a parameter used in high throughput screening assays to evaluate the quality and robustness of an assay, taking into account the assay window and standard deviations [29].

Small differences were obtained for the % Cytolysis across the different experiments, while the Z-factor ranged between 0.44 and 0.95 (S1 Fig). These values indicate that the assay is stable and can be potentially used for large screening campaigns and for comparison between plates.

**Fig 6:**
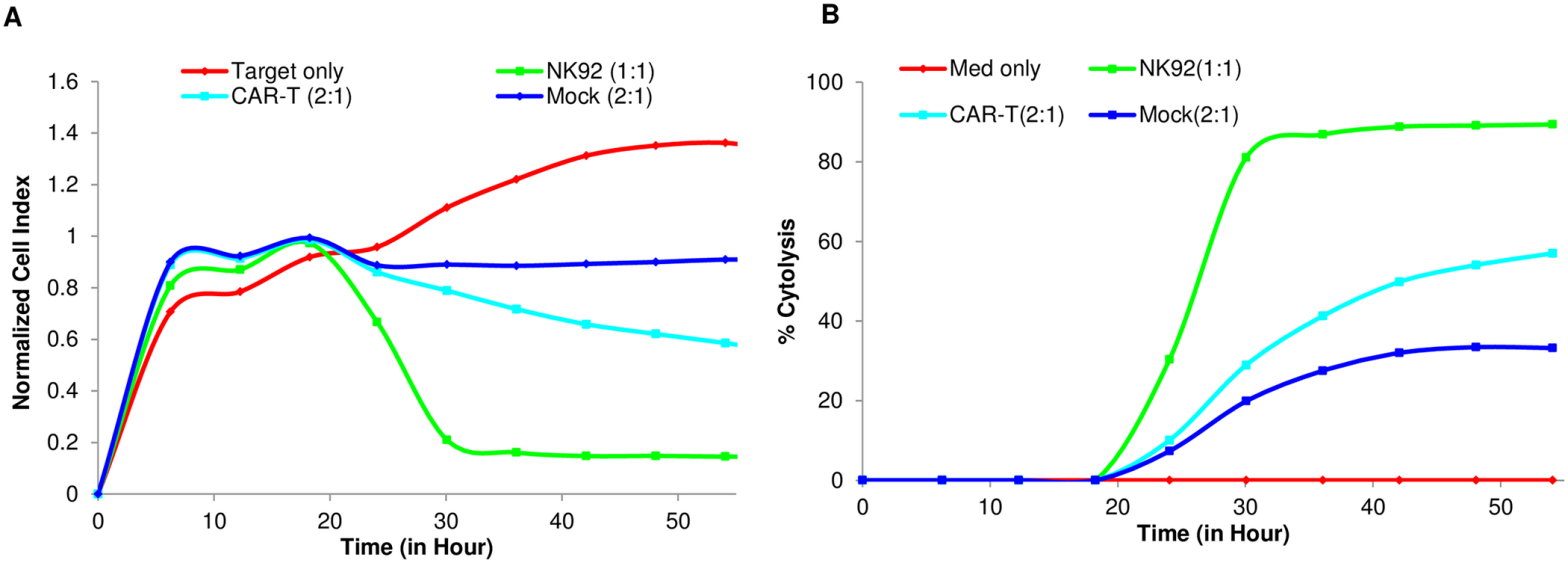
CAR-T mediated killing assay of tethered Raji B cells. (A) Raji cells were seeded at 40,000 cells per well on E plate. One day after seeding, effector CAR-T cells were added to the well at E:T ratio of 2:1. Mock CAR-T cells and NK92 were added for comparison. (B) Same data from (A) but displayed as % cytolysis.

In order to validate the xCELLigence assay for monitoring CAR-T cell potency, we treated CD40-tethered Raji cells with either CD19 targeting CAR-T (CD19-CAR-T) cells or mock T cells at different E:T ratios (E:T = 6:1, 5:1, 4:1,3:1 and 2:1). Fig 6A and 6B show real time impedance traces and % Cytolysis plots for Raji killing by CD19-CAR-T at an E:T 2:1. For purposes of comparison, killing by NK-92 at E:T of 1:1 is also shown. Interestingly, at all E:T ratios tested, both the CD19-CAR-T cells and mock-T cells display considerable cytolytic activity against Raji cells, though the difference between these effectors increases at lower E:T ratios (data not shown). To increase the sensitivity of this assay it may be necessary to further titrate the numbers of CD19-CAR-T and mock-T cells to find the optimal ratio where there is minimal cytolysis with mock-T cells and still significant cytolysis activity with CD19-CAR-T cells.

In addition to Raji cells, the above antibody tethering approach has been successfully used with other cancer cell lines like like Ramos, Daudi, and K562 cells (data not shown). In summary, the data shows that an antibody tethering approach can be used to specifically immobilize suspension liquid tumor cells on E-Plate electrodes, achieving a relevant signal to background ratio and enabling real-time cytolysis monitoring across different types of effectors.

### Real-time potency assay for assessment of immune checkpoint inhibitors

Function-blocking antibodies such as anti-PD-1 and anti-PDL-1, which disrupt the interaction of immune checkpoints on the surface of T cells and their cognate ligands on tumor cells, have shown considerable efficacy in the clinic against a number of cancers including melanomas, renal cancer and lung cancer [30–33]. It is therefore of interest to establish the usefulness of impedance monitoring for assessment of the effectiveness of immune checkpoint inhibitors.

**Fig 7:**
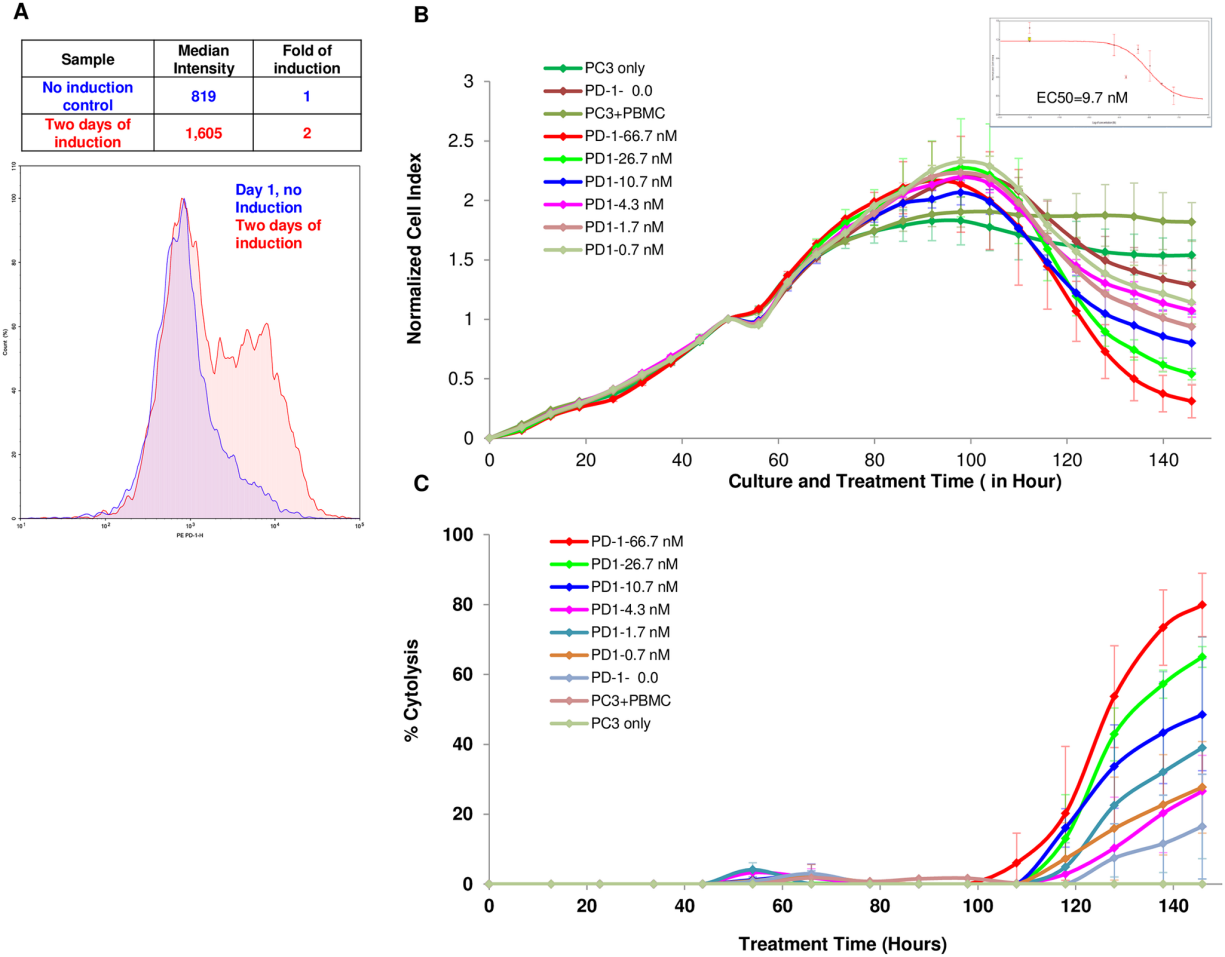
PC3 killing by PBMCs is enhanced by anti PD-1 blocking antibody. (A) Freshly isolated PMBCs shows increased PD-1 expression after SEB stimulation. (B) PC3 at 5,000 cells per well were seeded on E plate and treated with freshly isolated PBMCs two days after initial seeding in presence with increasing concentrations of the anti PD-1 blocking antibody. PC3 cells only and PC3 cells plus PBMCs and without antibody were included as controls. The insert show the dose dependent curve and EC_50_ calculated at t = 150 hours. (C) Same data from (B) but displays as % cytolysis.

To ensure adequate immune checkpoint expression, PBMCs were treated with the bacterial superantigen SEB to stimulate T cells and induce expression of PD-1 (Fig 7A). In order to assess the dose dependency of anti-PD1 immune checkpoint inhibitor, PC3 cells were seeded at 5000 cells/well in an E-Plate and 48 hours later were treated with SEB stimulated PBMCs at an E:T ratio of 8:1 in the presence of increasing concentrations of anti—PD1 antibody. As shown in Fig 7B and 7C, although PBMCs and SEB-stimulated PBMCs only slightly increased the extent of cytolysis of PC3 cells, the presence of anti-PD1 immune checkpoint inhibitor led to a significant dose-dependent increase in the extent of PBMCs-mediated cytolysis of PC3 cells. The efficacy of this anti-PD1 antibody was calculated from a dose-response curve (embedded in Fig 7B), with the EC_50_ found to be 9.7 nM. In a separate experiment using PBMCs from different donor, a similar dose dependency was observed and the EC_50_ found to be 2.9 nM (data not shown). In summary, the data described here demonstrates that the same real-time impedance-based technology described for other immunotherapeutic approaches can also be utilized for quantitative assessment of immune checkpoint inhibitors.

## Discussion

The arsenal of new and cutting edge therapies that aims to unmask the exquisite specificity and potency of the immune system against various types of cancers has greatly expanded over the course of the past several years. The regulatory approval of immune checkpoint inhibitors, BiTEs, oncolytic viruses, and the recent approval of autologous CAR-T therapy targeting ALL B cell leukemia has ushered in a new era of cancer therapy [34]. While the underlying mechanism of each of these therapies can vary significantly, the ultimate aim is to utilize the specificity and potency of the immune system to target cancerous cells while minimizing potentially harmful side effects. As such, the need for robust and sensitive in vitro potency assays that can provide incisive information during the R&D as well as manufacturing phases of such reagents is important. Potency assays are the key tool for evaluating the critical quality attributes (CQA) of immunotherapy products early in the development stages. An important criterion for potency assays is that they should take into consideration the mechanism of action of the product of interest, with the rationale that the more direct the potency assay mimics the mechanism of action, the more valuable and accurate the provided results will be [3, 5].

In this manuscript we describe the design and validation of a real-time potency assay and accompanying software that quantifies the cytolytic activity of immune cells towards target tumor cells using an impedance-based approach. In this xCELLigence assay target tumor cells adhere, either on their own or through the use of tethering antibodies, to the surface of interdigitated gold microelectrodes which are embedded in the bottom of microtiter plates (E-Plates). The interaction of the target tumor cells with the gold sensors generates an impedance signal which is reflective of the target cells number, size, and attachment strength [24]. A simplifying feature of this system is that the effector immune cells are not naturally adherent; and therefore generate a minimal impedance signal which can be easily subtracted. However, when immune cells attack and kill tumor cells through cytolysis, the viability of the target tumor cells reflected by real-time changes in impedance, providing a kinetic assessment of killing, a cytolysis process of target cells by immune cells. One of the major advantages of the xCELLigence potency assay described here is that it is non-invasive and precludes the use of fluorescent and radioactive labels, thereby providing greater flexibility in the choice of cells used and the duration of the experiment.

Using various commercially available immune effector cell lines such as NK-92 and TALL-104, together with adherent target tumor cell lines, we developed a sensitive and quantitative real-time potency assay based on cytolysis. Importantly, we validated the assay using a parallel image-based assay, demonstrating that the target cell lines are indeed killed in a time-dependent manner and that the residual impedance signal is proportional to the number of cells that are still adhering to the plate (Fig 1D). A critical attribute of the real-time potency assay described here is that its high sensitivity enables very low E:T ratios to be used (Figs 2 and 5). This minimizes non-specific killing and at the same time closely mimics serial killing of tumor cells, i.e. the ability of one immune effector cell to kill multiple tumor cells. In order to assess the extent of cytolysis easily and directly, the xCELLigence software includes an algorithm to convert Cell Index (CI) values to percent cytolysis. Furthermore, taking advantage of the instruments ability to track cytolysis in real-time, we introduced the KT_50_ parameter, or the time it takes to kill 50% of the tumor cells. This two dimensional parameter allows cytolysis comparison across the temporal scale, an aspect often overlooked with other end point assays.

In order to further validate the assay, we performed a parallel flow cytometry analysis measuring the extent of correlation between impedance reduction and the extent of apoptosis of target cells. Our data shows very good correlation and early sensitivity that is comparable to annexin V staining, which is one of the earliest indicators for apoptosis along with other markers(Figs 3 and 5).

We further tested the utility of the real-time potency assay using freshly isolated PBMCs in combination with BiTEs antibody directed against EpCAM, a known marker for various cancers of epithelial origin (Fig 4). The inclusion of 1 ug/mL BiTE in the assay facilitated the extent of cytolysis at different E:T ratios of PBMCs. While higher E:T ratios (10:1) displayed enhanced cytolytic activity in the presence of BiTE antibodies, the dose-dependency of BiTE antibodies was more apparent when lower E:T ratio of PBMCs (1.25:1) was used, suggesting that when optimizing assay conditions, multiple E:T ratios should be tested to obtain optimal and accurate dose-response of the BiTE antibody.

In order to extend the range of applications, we adapted the real-time potency assays to liquid tumor cell lines which are typically suspension cells. A tethering approach was developed (Fig 5A), where an antibody that is specific for a target expressed on suspension tumor cells but not the effector cells is utilized to selectively tether target B cell lines to the surface of the gold sensors embedded in the bottom of the E-Plates. Since the expression level of the selective markers vary in different target cell lines, the amount and specificity of tethering antibody should be optimized prior to performing the assay. Raji cells as well as several other suspension cells such as Daudi and Ramos were successfully tethered using this approach. As shown in Figs 5 and 6, both NK92 and CD19-CAR-T cells were able to kill the target Raji cells in a density-dependent manner. For CAR-T mediated killing, the use a mock-transfected T cells as control accounted for the extent of non-specific killing. Reducing the E:T ratio and extending the time of the assay can insure more selective cytolysis by the target-specific CAR-T.

Checkpoint inhibitors, either alone or in combination with other inhibitors or small molecules, have shown considerable efficacy in the clinic. We tested the ability of the real-time impedance-based assay to assess the potency of checkpoint inhibitors in the context of PBMCs stimulated with the bacterially derived super-antigen (SEB) to increase the expression of PD1 on the surface of the immune cells [30–33, 35–37]. Increasing the concentration of anti-PD1 antibody in the presence of fixed E:T ratio of PBMCs to PC3 cells dose-dependently increased the extent of PBMCs-mediated cytolysis (Fig 7), demonstrating the feasibility of using the methodology to screen for combination therapies which enhance the extent of immune cell-mediated cytolysis of tumor cells.

In summary, the real-time impedance-based platform and reagents described in this paper provides a sensitive, robust and reproducible assay system that can be used for quantitative assessment of different immunotherapy approaches in vitro [38–41]. Despite the fact that various groups have already incorporated this technology for assessment of BiTEs and bispecific antibodies [25, 26] oncolytic viruses [42, 43], NK-mediated cytolysis [44, 45] and for assessment of engineered T cells such as CAR-T [10, 46, 47], the new adaptation to suspension cancer cells and the companion immunotherapy analysis software promises to further extend and accelerate the adoption of the technology for immunotherapy approaches. While the assay can be used during the research and development phases of different immunotherapies, it can also be incorporated as part of the manufacturing process for batch release of different types of immunotherapy reagents. Also, we have demonstrated the reproducibility and robustness of the assay for possible application as QC potency assay in manufacturing and for large screening campaigns (S1 Fig). Furthermore, the assay can also be potentially used with primary cancer cells [48–50] and adapted for in vitro clinical testing using the patient’s own tumor primary cells along with immune cells harvested from the same patient or from donors.

## Supporting information

S1 Fig Reproducibility and Z-factor for three independent experiments. (A) One representative plate data of the Raji cells killed by NK92 cells. Red labeled wells indicate Raji target cells seeded at a density of 30,000 cells per well. Green labeled wells indicate Raji target cells where NK92 was added at E:T ratio of 1:1. Blue labeled wells indicate Raji cells with added NK92 at E:T ratio of 0.1:1. Pink labeled wells indicate Raji cells treated with cytolysis buffer. (B) Top is diagram of the plate map which was set up in 3 replicates for the purposes of Z-factor calculation. The color representation of the wells is similar to those described in A. The white colored wells represent key control wells where NK92 only is added in high and low density and also a medium only control. These last two types of controls are used by the xIMT software to subtract residual background signal during the % cytolysis calculation. Bottom tables are parameter of % cytolysis Z-factor values calculation of these three independent experiments at 4 and 24 hours after NK92 addition. (C) Normalized Cell Index curves of these key controls along with the target only control (red). NK high density only is in light green, NK low density only is in purple and light blue is for medium only.
(TIF)

S1 Data Support information for Figs 1–7. (XLSX)

## Abbreviations

ANOVA: A collection of statistical models for analysis of the variance
BiTE: Bispecific T cell Engagers
CAR-T: chimeric antigen receptor-T cells
CI: Cell Index
E:T Ratio: Effector versus Target cells ratio
E-Plate: A special plate with electronic sensor on the well bottom to be used in the RTCA system
KT_50_: the time to kill50% of target tumor cells
RTCA: Real Time Cell Analyzer
SEB: *Staphylococcus* enterotoxin B
xIMT: brand name for the software to operate RTCA system and analyze cytolysis data
Z-factor: A measurement of the statistical effect size to assess high throughput assay quality
