# Supplementary figures and images for: In vitro immunotherapy potency assays using real-time cell analysis
Source: PLoS One. 2018 Mar 2;13(3):e0193498. doi: 10.1371/journal.pone.0193498 (PMC5834184; doi:10.1371/journal.pone.0193498)

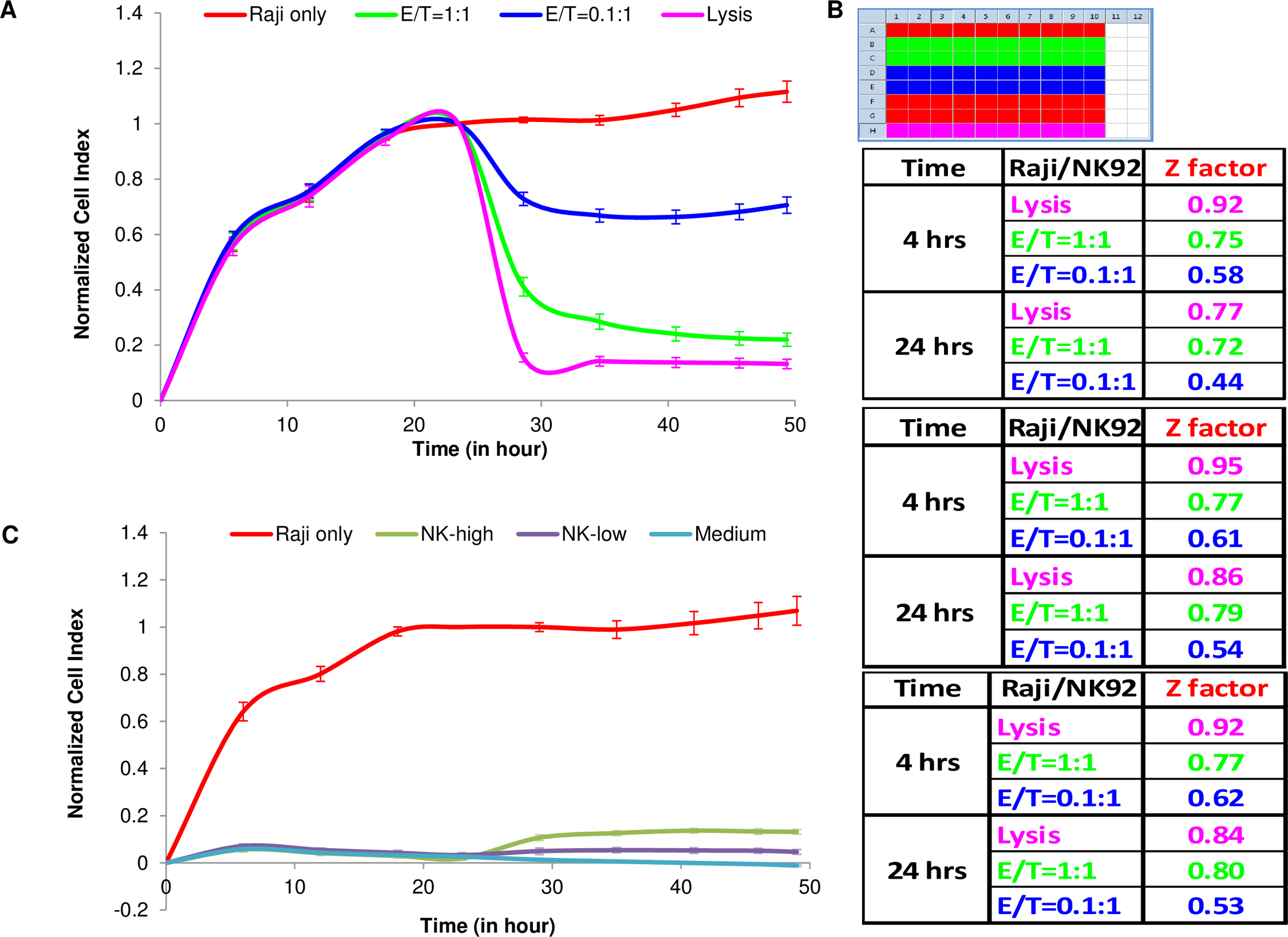

Supplement: S1 Fig — (A) One representative plate data of the Raji cells killed by NK92 cells. Red labeled wells indicate Raji target cells seeded at a density of 30,000 cells per well. Green labeled wells indicate Raji target cells where NK92 was added at E:T ratio of 1:1. Blue labeled wells indicate Raji cells with added NK92 at E:T ratio of 0.1:1. Pink labeled wells indicate Raji cells treated with cytolysis buffer. (B) Top is diagram of the plate map which was set up in 3 replicates for the purposes of Z-factor calculation. The color representation of the wells is similar to those described in A. The white colored wells represent key control wells where NK92 only is added in high and low density and also a medium only control. These last two types of controls are used by the xIMT software to subtract residual background signal during the % cytolysis calculation. Bottom tables are parameter of % cytolysis Z-factor values calculation of these three independent experiments at 4 and 24 hours after NK92 addition. (C) Normalized Cell Index curves of these key controls along with the target only control (red). NK high density only is in light green, NK low density only is in purple and light blue is for medium only. (TIF) [file pone.0193498.s001.tif]
